# Supplementary material for: BAF complexes maintain accessibility at stimulus-responsive chromatin and are required for transcriptional stimulus responses
Source: bioRxiv. 2026 Mar 21:2026.03.19.712964. Preprint. [Version 1] doi: 10.64898/2026.03.19.712964 (PMC13015267; doi:10.64898/2026.03.19.712964)
Supplement: 1 — Supplemental Figure 1 (Related to Figure 1): Pharmacological disruption of BAF activity results in widespread accessibility changes, with outsize effects at enhancers. (A) BRM014 treatment for 24 h does not significantly affect cell viability. The shows viability data with a restricted y-axis range. P-values were calculated using Student’s t-tests. n.s., not significant (B) Accessibility changes (from ATAC-seq) due to BAFinh are strongly correlated across timepoints and doses. (C) Treatment with ACBI1, a proteolysis-targeting chimera against SMARCA2/A4, leads to undetectable SMARCA4 and strongly reduced SMARCA2 after 24h of treatment. A representative Western blot is shown. (D) Representative genome browser examples demonstrating similar effects of BAFinh and ACBI1 at enhancers (left; decreased accessibility) and promoters and insulators (right; maintained accessibility). (E) Accessibility changes induced by BAFinh and ACBI1 are strongly correlated. P-value calculated using a two-sided Pearson correlation test. (F) Enrichment of histone modifications and TSS enrichment between relevant ChromHMM states, adapted from Ernst et al., 2011 [25]. ChromHMM states used to define promoters, enhancers, and insulators are indicated at left. (G) Enhancer-associated ChromHMM states are enriched for BAF-dependent chromatin accessibility while promoter-and insulator-associated states are depleted. (H) Among cRE classes, enhancers show the highest proportion of significantly BAF-dependent elements across both BAFinh dose and duration. (I) Using ENCODE candidate cis-regulatory element (cCRE) annotations [26], distal enhancer-like signature (dELS) cREs exhibit significantly greater accessibility loss upon BAFinh compared to promoter-like signature (PLS) and CTCF-only elements. (J) Alternative cRE annotation scheme based on TSS proximity and ChIP–seq peak overlap (left). Enhancers defined using this approach similarly show greater loss of accessibility upon BAFinh relative to other cRE [file NIHPP2026.03.19.712964V1-supplement-1.pdf]

bioRxiv preprint doi: https://doi.org/10.64898/2026.03.19.712964; this version posted March 21, 2026. The copyright holder for this preprint (which was not certified by peer review) is the author/funder, who has granted bioRxiv a license to display the preprint in perpetuity. It is made available under aCC-BY 4.0 International license.

Supplemental Figure 1

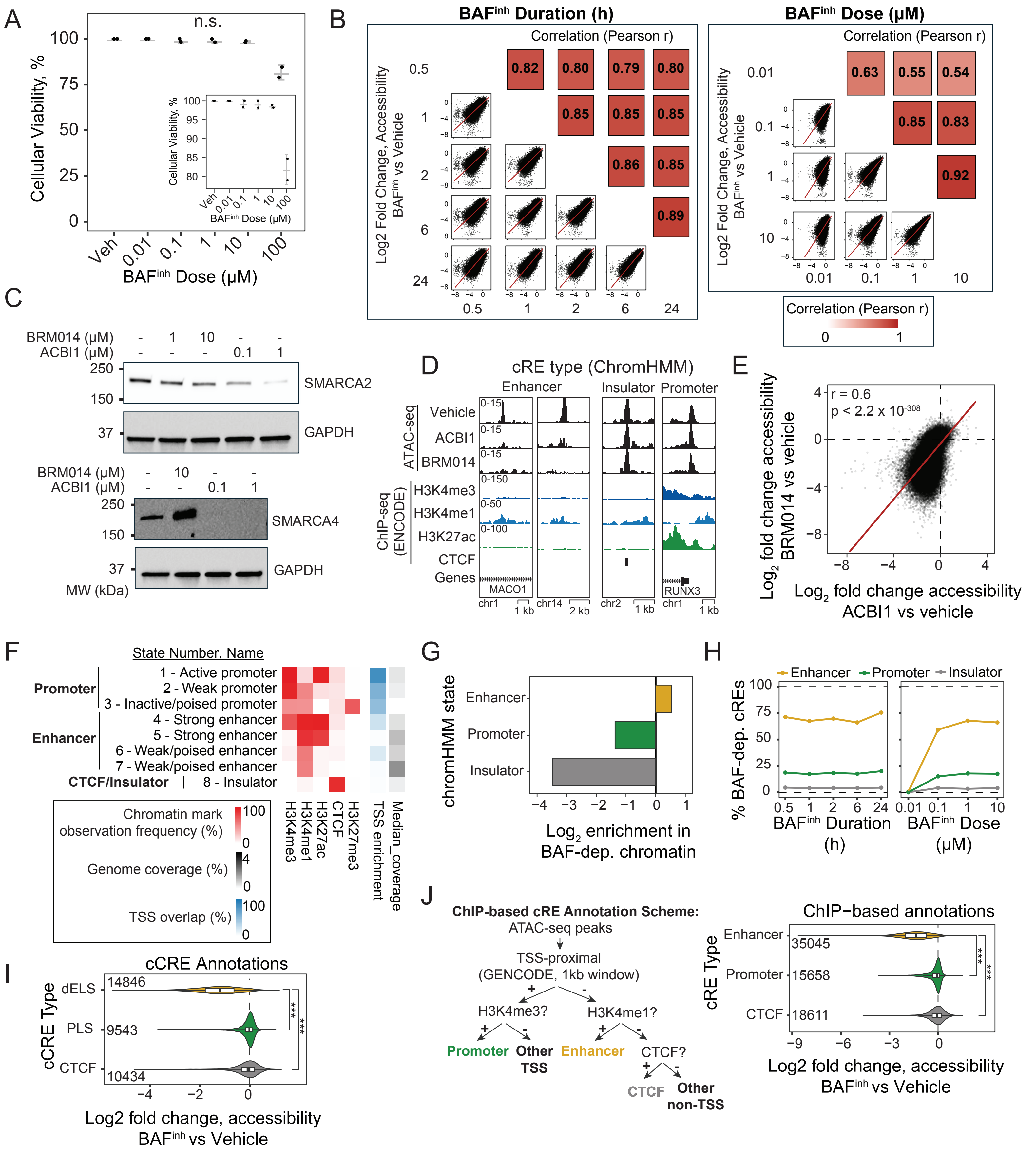

bioRxiv preprint doi: https://doi.org/10.64898/2026.03.19.712964; this version posted March 21, 2026. The copyright holder for this preprint (which was not certified by peer review) is the author/funder, who has granted bioRxiv a license to display the preprint in perpetuity. It is made available under aCC-BY 4.0 International license.

Supplemental Figure 1 (Related to Figure 1): Pharmacological disruption of BAF activity results in widespread accessibility changes, with outsize effects at enhancers. (A) BRM014 treatment for 24 h does not significantly affect cell viability. The shows viability data with a restricted y-axis range. P-values were calculated using Student's t-tests. n.s., not significant (B) Accessibility changes (from ATAC-seq) due to BAF^{inh} are strongly correlated across timepoints and doses. (C) Treatment with ACBI1, a proteolysis-targeting chimera against SMARCA2/A4, leads to undetectable SMARCA4 and strongly reduced SMARCA2 after 24h of treatment. A representative Western blot is shown. (D) Representative genome browser examples demonstrating similar effects of BAF^{inh} and ACBI1 at enhancers (left; decreased accessibility) and promoters and insulators (right; maintained accessibility). (E) Accessibility changes induced by BAF^{inh} and ACBI1 are strongly correlated. P-value calculated using a two-sided Pearson correlation test. (F) Enrichment of histone modifications and TSS enrichment between relevant ChromHMM states, adapted from Ernst et al., 2011 [25]. ChromHMM states used to define promoters, enhancers, and insulators are indicated at left. (G) Enhancer-associated ChromHMM states are enriched for BAF-dependent chromatin accessibility while promoter-and insulator-associated states are depleted. (H) Among cRE classes, enhancers show the highest proportion of significantly BAF-dependent elements across both BAF^{inh} dose and duration. (I) Using ENCODE candidate cis-regulatory element (cCRE) annotations [26], distal enhancer-like signature (dELS) cREs exhibit significantly greater accessibility loss upon BAF^{inh} compared to promoter-like signature (PLS) and CTCF-only elements. (J) Alternative cRE annotation scheme based on TSS proximity and ChIP–seq peak overlap (left). Enhancers defined using this approach similarly show greater loss of accessibility upon BAF^{inh} relative to other cRE classes. ***, $p < 2.2 \times 10^{-308}$**,** Wilcoxon rank-sum test.

bioRxiv preprint doi: https://doi.org/10.64898/2026.03.19.712964; this version posted March 21, 2026. The copyright holder for this preprint (which was not certified by peer review) is the author/funder, who has granted bioRxiv a license to display the preprint in perpetuity. It is made available under aCC-BY 4.0 International license.

Supplemental Figure 2

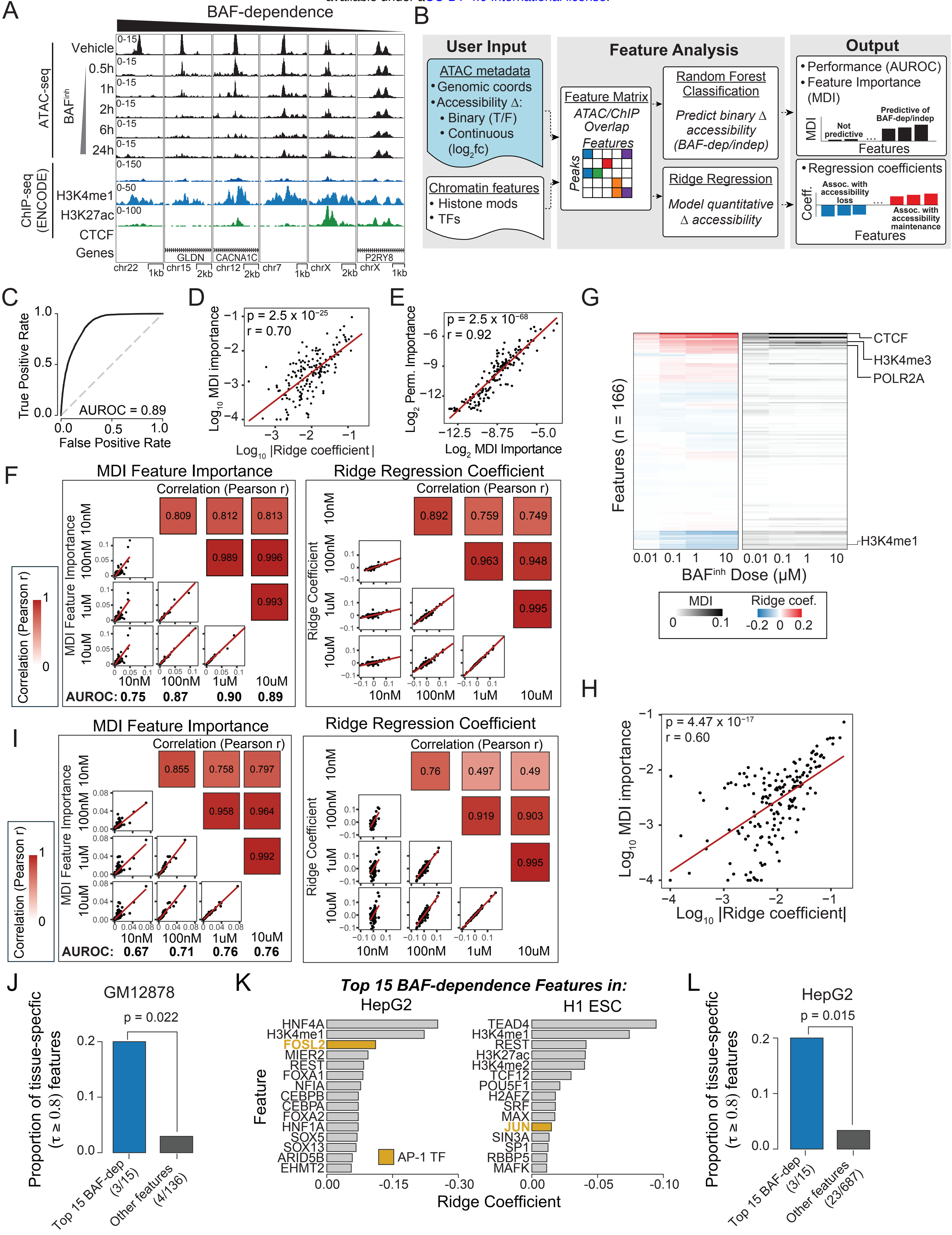

bioRxiv preprint doi: https://doi.org/10.64898/2026.03.19.712964; this version posted March 21, 2026. The copyright holder for this preprint (which was not certified by peer review) is the author/funder, who has granted bioRxiv a license to display the preprint in perpetuity. It is made available under aCC-BY 4.0 International license.

Supplemental Figure 2 (Related to Figure 2): Robust performance and consistent feature associations in machine learning models predicting BAF-dependent accessibility. (A) Genome browser examples demonstrating heterogeneity in enhancer responses to BAF^{inh}. (B) Overview of the machine learning approach. ENCODE TF and histone ChIP-seq experiments are filtered by experiment quality (FRiP score – see **Methods**) and intersected with consensus ATAC-seq peaks to construct a feature matrix, which is then used to model binary BAF dependence (random forest classification) and continuous accessibility changes (ridge regression). (C) Discriminative performance of the random forest model when applied to all cis-regulatory elements (cREs), assessed by AUROC. (D) Feature importance metrics derived from random forest (mean decrease in impurity) and ridge regression (regression coefficients) are highly correlated, demonstrating agreement between approaches. (E) MDI and permutation-based feature importances are strongly correlated. P-value calculated using two-sided Pearson correlation test. (F) Feature importance metrics from all-cRE models trained using different BAF^{inh} doses are highly correlated, indicating consistency of feature associations. (G) All-cRE models identify expected features associated with BAF-dependence (e.g., H3K4me1) and -independence (e.g., H3K4me3, Pol2, CTCF). (H) MDI feature importance and ridge regression coefficients calculated from an enhancer-only feature matrix are significantly correlated. (I) Feature importance metrics from enhancer-only models using different BAF^{inh} doses are highly concordant. (J) Features associated with BAF dependence are significantly enriched for tissue-restricted gene expression ($\tau > 0.8$) compared to other features. P-value calculated using Fisher's exact test; numbers of tissue-restricted and non-restricted features for each category are indicated (K) AP-1 TFs are represented among the top BAF-dependence features in both HepG2 and H1 embryonic stem cells. (L) In HepG2 cells, BAF-dependence features are significantly enriched for tissue-restricted expression. P-value calculated using Fisher's exact test; numbers of tissue-restricted and non-restricted features for each category are indicated.

bioRxiv preprint doi: https://doi.org/10.64898/2026.03.19.712964; this version posted March 21, 2026. The copyright holder for this preprint (which was not certified by peer review) is the author/funder, who has granted bioRxiv a license to display the preprint in perpetuity. It is made available under aCC-BY 4.0 International license.

Supplemental Figure 3

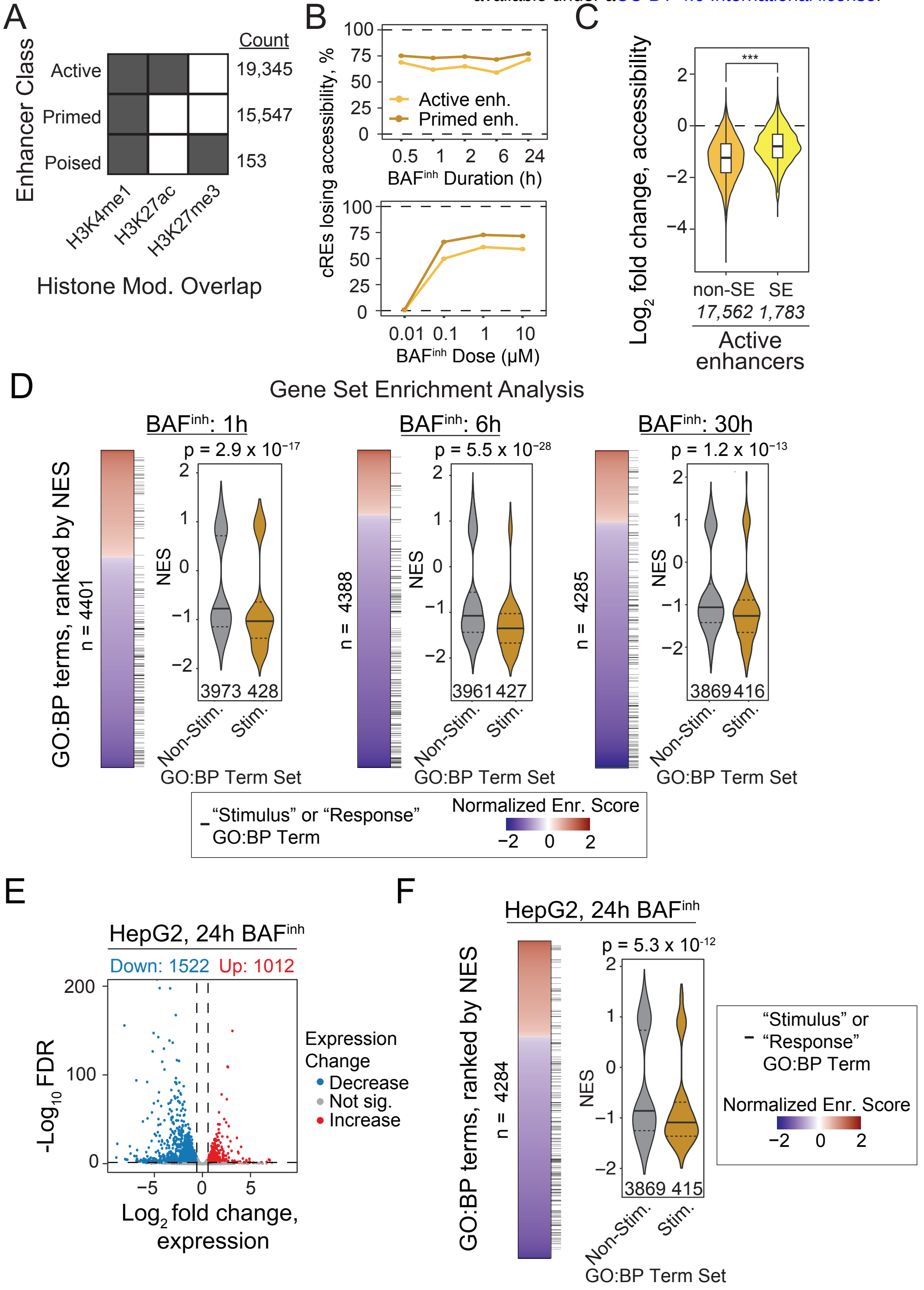

Supplemental Figure 3 (Related to Figure 3): BAFinh preferentially disrupts primed enhancers and stimulus-responsive transcriptional pathways. (A) Enhancers were classified as active, primed, or poised based on overlaps with H3K27ac and H3K27me3 ChIP-seq peaks. (B) Primed enhancers show a higher proportion of significantly BAF-dependent elements compared to active enhancers across both BAFinh duration (top) and dose (bottom). (C) Active enhancers within super-enhancers (SEs) show reduced BAF-dependence compared with non-SE active enhancers. (D) Genes associated with stimulus–response pathways are downregulated following BAFinh in GM12878 cells. Gene Ontology Biological Process (GO:BP) terms are ranked by Normalized Enrichment Scores (NES) from GSEA performed for 1h, 6h, and 30h BAFinh, displayed as heatmaps, and categorized based on whether term names contained "stimulus" or "response" (black bars). Violin plots show distributions of NES scores for stimulus-and non-stimulus term sets, where solid lines represent median NES and dashed lines represent 25th and 75th percentiles. P-values were calculated using one-sided Wilcoxon rank-sum tests; total numbers of GO:BP terms analyzed for each timepoint as well as sizes of stimulus and non-stimulus term sets are indicated. (E) Transcriptional changes upon BAFinh (10μM, 24h) in HepG2 cells. (F) As in GM12878, stimulus-associated pathways are downregulated upon BAFinh in HepG2. P-value calculated as in (D).

bioRxiv preprint doi: https://doi.org/10.64898/2026.03.19.712964; this version posted March 21, 2026. The copyright holder for this preprint (which was not certified by peer review) is the author/funder, who has granted bioRxiv a license to display the preprint in perpetuity. It is made available under aCC-BY 4.0 International license.

Supplemental Figure 4

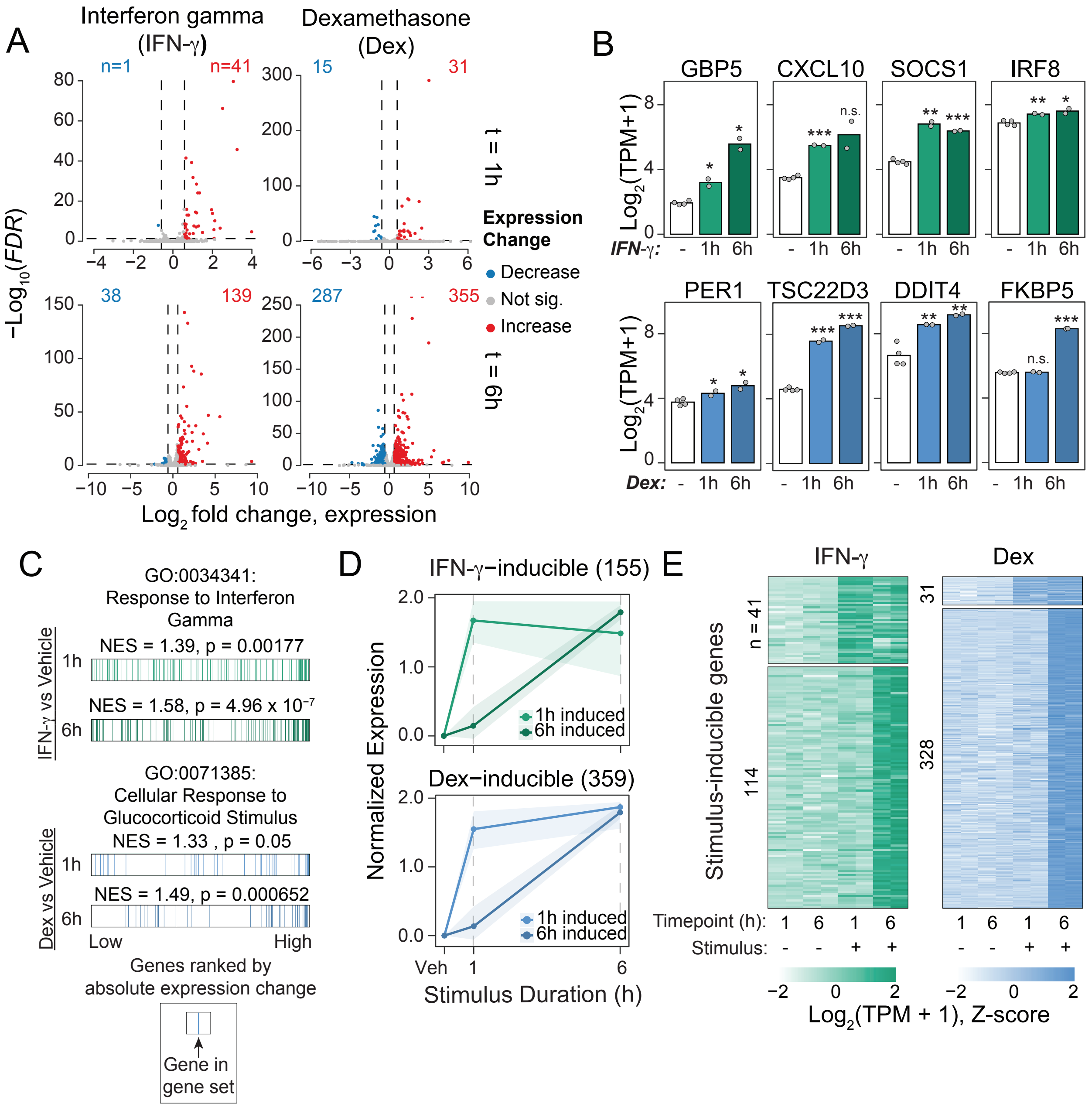

Supplemental Figure 4 (Related to Figure 4): Time-dependent transcriptional responses to IFN-γ and dexamethasone in GM12878 cells. (A) Volcano plots showing the magnitude and direction of transcriptional responses to IFN-γ and dexamethasone (Dex) at 1 h and 6 h. Numbers of genes with significantly increased (FDR < 0.05, log2FC > log2(1.5)) or decreased (FDR < 0.05, log2FC < -log2(1.5)) expression are indicated. (B) Representative canonical IFN-γ– and Dex-induced genes, illustrating stimulus-specific transcriptional induction. P values were calculated using a Student's t-test (*, $p < 0.05$; **, $p < 0.01$, ***, $p < 0.001$). (C) Specificity of transcriptional responses to IFN-γ and Dex demonstrated by gene set enrichment analysis (GSEA) on IFN-γ response and glucocorticoid response GO:BP terms, respectively. (D) Median expression trajectories of 1h- and 6h-induced genes following IFN-γ or Dex treatment. Expression is shown as Z-scored log2(TPM + 1), normalized to vehicle. Shaded regions denote the interquartile range. (E) Heatmap showing gene-level expression changes for early- and late-induced genes in response to IFN-γ and Dex.

bioRxiv preprint doi: https://doi.org/10.64898/2026.03.19.712964; this version posted March 21, 2026. The copyright holder for this preprint (which was not certified by peer review) is the author/funder, who has granted bioRxiv a license to display the preprint in perpetuity. It is made available under aCC-BY 4.0 International license.

Supplemental Figure 5

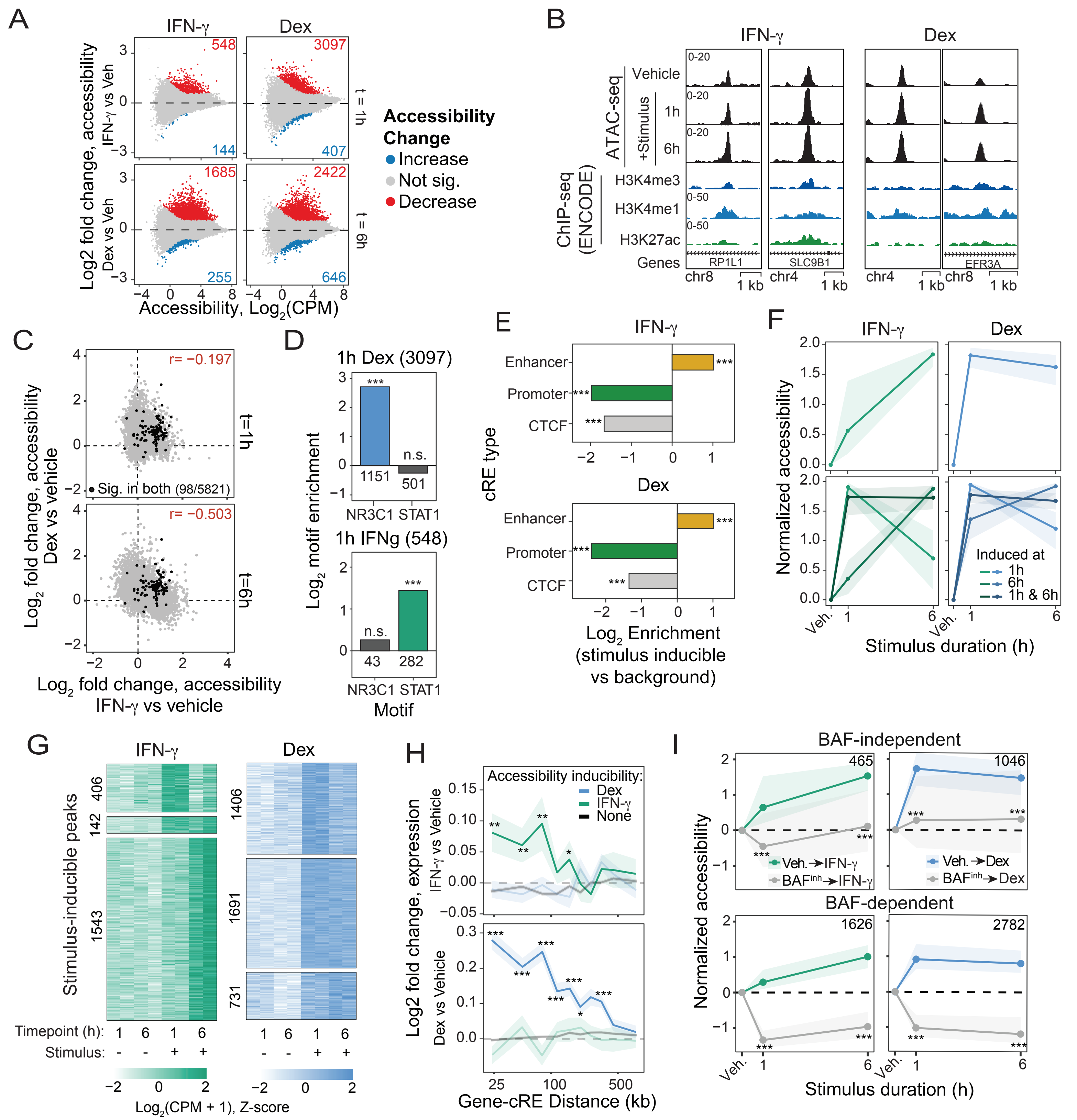

bioRxiv preprint doi: https://doi.org/10.64898/2026.03.19.712964; this version posted March 21, 2026. The copyright holder for this preprint (which was not certified by peer review) is the author/funder, who has granted bioRxiv a license to display the preprint in perpetuity. It is made available under aCC-BY 4.0 International license.

Supplemental Figure 5 (Related to Figure 5): Chromatin accessibility changes in response to IFN-γ and dexamethasone in GM12878 cells. (A) MA plots showing chromatin accessibility changes in response to IFN-γ or Dex at 1 h and 6 h. (B) Representative genome browser examples of enhancers that gain accessibility in response to IFN-γ (left) or Dex (right). (C) Chromatin accessibility gains are stimulus specific. All cREs showing significant induction in either stimulus or time point are plotted by log2 fold change at 1 h (top) or 6 h (bottom); cREs significantly induced by both stimuli are indicated. (D Enriched transcription factor motifs at 1h stimulus-inducible cREs correspond to the applied stimulus. Numbers of inducible loci containing each motif are indicated. P values were calculated using Fisher's exact tests (E) Stimulus-inducible cREs are enriched for enhancer annotations relative to background regions. P-values were calculated using Fisher's exact tests (F) Median accessibility gains at all inducible regions (top) and separated by induction timepoint (1h-only, both timepoints, 6h-only). Median normalized accessibility (Z-scored log2(CPM+1), normalized to vehicle) is shown, with the shaded region representing the interquartile range. (G) For each IFN-γ (left) or Dex (right) inducible cRE, normalized accessibility levels are shown, separated by induction timepoint. cREs are grouped based on the timepoint(s) at which significant stimulus-responsive accessibility gains are observed: 1h-only (top), both timepoints (middle) and 6h-only (bottom). (H) Genes linked by promoter capture Hi-C (PCHi-C) to stimulus-inducible cREs exhibit stimulus-specific transcriptional responses. Genes linked to IFN-γ-inducible, Dex-inducible, or non-inducible cREs were binned by contact distance, and mean expression fold change in response to IFN-γ (top) or Dex (bottom) was calculated for each bin. Values are plotted as mean fold change (line) and standard error of the mean (shaded region). P values were calculated using Wilcoxon rank-sum tests (IFN-γ vs Dex) for each decile and adjusted for multiple testing using the Benjamini-Hochberg method). (I) After stratifying stimulus-inducible cREs by basal BAF dependence, both BAF-dependent and BAF-independent categories show reduced accessibility induction following BAF^{inh} pretreatment. Normalized accessibility and IQR is shown as in (G). Numbers of cREs in each category for each stimulus are indicated. P-values were calculated from Wilcoxon rank-sum tests. *, $p < 0.05$; **, $p < 0.01$; ***, $p < 0.001$; n.s., not significant.
